# Immunological Risk Factors in Recurrent Pregnancy Loss: Guidelines Versus Current State of the Art

**DOI:** 10.3390/jcm10040869

**Published:** 2021-02-20

**Authors:** Kilian Vomstein, Katharina Feil, Laura Strobel, Anna Aulitzky, Susanne Hofer-Tollinger, Ruben-Jeremias Kuon, Bettina Toth

**Affiliations:** 1 Department of Gynecological Endocrinology and Reproductive Medicine, Medical University Innsbruck, Anichstrasse 35, 6020 Innsbruck, Austria; katharina.feil@tirol-kliniken.at (K.F.); laura.strobel@student.i-med.ac.at (L.S.); anna.aulitzky@i-med.ac.at (A.A.); susanne.tollinger@tirol-kliniken.at (S.H.-T.); bettina.toth@i-med.ac.at (B.T.); 2 Department of Gynecological Endocrinology and Fertility Disorders, Ruprecht-Karls University Heidelberg, Im Neuenheimer Feld 440, 69120 Heidelberg, Germany; ruben.kuon@med.uni-heidelberg.de

**Keywords:** recurrent miscarriage, reproductive immunology, NK cells, regulatory T cells

## Abstract

Around 1–5% of all couples experience recurrent pregnancy loss (RPL). Established risk factors include anatomical, genetic, endocrine, and hemostatic alterations. With around 50% of idiopathic cases, immunological risk factors are getting into the scientific focus, however international guidelines hardly take them into account. Within this review, the current state of immunological risk factors in RPL in international guidelines of the European Society of Reproduction and Embryology (ESHRE), American Society of Reproductive Medicine (ASRM), German/Austrian/Swiss Society of Obstetrics and Gynecology (DGGG/OEGGG/SGGG) and the Royal College of Obstetricians and Gynecologists (RCOG) are evaluated. Special attention was drawn to recommendations in the guidelines regarding diagnostic factors such as autoantibodies, natural killer cells, regulatory T cells, dendritic cells, plasma cells, and human leukocyte antigen system (HLA)-sharing as well as treatment options such as corticosteroids, intralipids, intravenous immunoglobulins, aspirin and heparin in RPL. Finally, the current state of the art focusing on both diagnostic and therapeutic options was summarized.

## 1. Introduction

The WHO defines recurrent pregnancy loss (RPL) as 3 or more consecutive pregnancy losses before the 20th week of pregnancy, while the American Society for Reproductive Medicine (ASRM) defines RPL after two pregnancy losses with clinical evidence of pregnancy (sonographic or histopathological evidence of pregnancy) [1,2]. About 1–5% of couples are affected by RPL, with significant consequences concerning their partnership and quality of life [3]. In the last years, the European Society of Reproduction and Embryology (ESHRE) [4], ASRM [5], German/Austrian/Swiss Society of Obstetrics and Gynecology (DGGG/OEGGG/SGGG) [6] and the Royal College of Obstetricians and Gynecologists (RCOG) [7] have developed guidelines to define a diagnostic and therapeutic work-up in RPL patients. In detail, the guidelines were published between 2011 and 2018. The RCOG recommendations from 2011 were updated in 2014 and 2017, the ARSM expert letter was updated in 2012. The ESHRE guideline was published in 2017. The first version of the DGGG/OEGGG/SGGG guideline was published in 2006, updated in 2013, and upgraded to a higher evidence-level (S2k) in 2018 and is currently under review. However, the diagnostic and therapeutic recommendations differ considerably—already regarding the definition of RPL.

All guidelines define several established risk factors for RPL: parental genetic disorders, uterine anatomical malformations, endocrine dysfunction, and hemostatic disorders [4,5,6,8].

However, regarding immunological disorders, RPL patients are currently only screened for an antiphospholipid syndrome, as recommended by all guidelines although after standardized diagnostic including all mentioned risk factors, 50% of RPL cases continue to remain elusive.

Today it is well known that the maternal immune system is not ignorant of the fetus, but it recognizes and responds to antigens from the developing embryo. Therefore, establishing the appropriate maternal tolerance towards the embryo is crucial for securing implantation and a successful pregnancy. Nevertheless, the exact mechanisms governing the physiology of tolerance at the feto-maternal interface remain poorly understood. Using several diagnostic methods, studies described immunological parameters that differ between RPL patients and healthy women, including: uterine and peripheral blood natural killer cells (uNK, pNK cells) [9,10,11,12], regulatory T-cells (Tregs) [13,14], and the human leukocyte antigen (HLA) profiling of affected couples [15]. While previous reviews compared the RCOG, ASRM, and ESHRE guidelines in general [16,17], this review aims to discuss current data on immunological disorders in RPL patients and to put them into perspective with current guidelines on RPL. Therefore, we comment on the above-mentioned guidelines with regard to recommended immunological diagnostics and therapies and provide an overview on the currently most promising immune alterations in RPL, such as auto-antibodies, NK-cells, Treg, dendritic cells (DC), plasma cells, as well as HLA and their therapeutic options. Furthermore, we finally summarize the current state of the art concerning diagnostic and therapy of immunologic risk factors in RPL.

## 2. Autoimmunity

An overview of autoimmune risk factors mentioned in the different guidelines is summarized in Table 1. Table 2 shows the autoimmune risk factors that according to the current state of the art should be evaluated.

### 2.1. Antinuclear Antibodies

There is no conclusive opinion on the clinical impact of antinuclear antibodies (ANA) in RPL: currently, no international guideline recommends routine testing for ANA, only the ESHRE guideline considers testing for explanatory purpose. There are four possible mechanisms that ANA might be involved in the pathogenesis of RPL: (1) A reduced oocyte quality and embryo development [18,19], (2) the activation of an intraplacental complement cascade [20], (3) the deposition of immune-complexes in placental tissue [20,21], and (4) the activation of plasmacytoid dendritic cell resulting in an increased production of inflammatory cytokines [22]. Previous studies showed both, increased as well as normal titres of ANA in RPL in comparison to controls [23,24]. Recently, a meta-analysis including 21 studies on ANA in RPL, reported a significantly higher rate of elevated ANA titres in RPL patients (22%) compared to controls (8.3%) as well as a significant association between positive ANA and a risk for RPL [25]. Especially with high titres (≥1:80; ≥1:160) or a homogenous ANA pattern, the association was more evident. Therefore, the best predictive cut-off level for ANAs in RPL patients remains to be defined [23,26,27]. The guideline of the DGGG/SGGG/OEGGG states, that if elevated ANA titres (referred to as “above lab reference range”) are diagnosed in RPL patients, the antibodies should be further differentiated (SS-A/RO and SS-B/lupus anticoagulant (LAC)antibodies) to rule out a Sjögren’s syndrome or lupus erythematosus (as a neonatal lupus syndrome could lead to a fetal AV block). As mentioned in Table 2 we recommend using 1:160 as a cut-off level for “elevated” ANA and for detailed analysis (further differentiation).

### 2.2. Antiphospholipid Syndrome

An anti-phospholipid syndrome (APLS) occurs in 5–20% of RPL patients and includes the definition of RPL already in its diagnostic criteria [28]. Recent studies indicate that the incidence of an APLS in RPL might be overestimated [29]. To diagnose an APLS, anti-phospholipid antibodies (anti-cardiolipin (ACA), LAC or anti-β 2-glycoprotein-1 (ß2GP) antibodies) must be detected twice in medium to high titres at an interval of 12 weeks. Furthermore, clinical criteria such as venous or arterial thrombosis, ≥3 pregnancy losses before the 10th week of pregnancy, ≥1 late pregnancy loss, or premature birth at <34 week of pregnancy due to placental insufficiency or preeclampsia must be present [30]. Obstetric anti-phospholipid syndrome (OAPLS) is considered to comprise the obstetric APLS criteria without previously presenting thrombotic episodes [31,32], as obstetric complications are one of the major manifestations in APLS. However, as many RPL patients fail to fulfill the diagnostic criteria, a non-criteria OAPLS (NC-OAPLS) is described [33,34,35]. To diagnose a NC-OAPLS (1) the combination of non-criteria clinical manifestations, such as late pre-eclampsia or two unexplained miscarriages, with international consensus laboratory criteria or (2) international consensus clinical criteria with a non-criteria laboratory manifestation, such as low positive ACA/ß2GP need to be present [35].

Recently, clinical and laboratory differences were shown between OAPLS and NC-OAPLS patients: double or triple positivity for anti-phospholipid antibodies were significantly more present in OAPLS patients, while single positivity was more frequent in NC-OAPLS RPL patients [36]. Although the fetal-maternal outcome was similar when treated, obstetric outcomes differ between both groups: OAPLS patients showed occurrence of one miscarriage, fetal loss, and stillbirth more often, while in NC-OAPLS patients ≥1 miscarriage was more frequent [36].

The higher risk for RPL in patients with an APLS is discussed to be due to an elevated risk of placental micro-thrombosis, as well as direct effects of the auto-antibodies on the trophoblast itself [37]. Besides the anti-phospholipid antibody-mediated hypercoagulable state, there is evidence for a more inflammatory cause in OAPLS: A complement activation resulting in a neutrophil and monocyte activation with release of reactive oxygen species, TNF-α, anti-angiogenic factors, and tissue factor is considered to lead to fetal injury and placental insufficiency [38,39]. A review of histopathologic alterations in placentae of women with APL antibodies showed a higher prevalence of placental infarction and decidual inflammation but fewer thrombotic signs such as arthrosis or intraluminal thrombosis [40]. If left untreated, an APLS results in a pregnancy loss in 50–90% of the cases, while live birth rates (LBR) of around 70% have been described with adequate treatment [41].

All guidelines recommend testing for IgG/IgM ACA, LAC; however, only the ESHRE, DGGG/OEGGG/SGGG, and ASRM recommend screening for ß2GP antibodies. In accordance with the DGGG/OEGGG/SGGG a “non-criteria APLS” should also be considered if clinical manifestations are present. These imply renal microangiopathy, neurological disorders, cardiac manifestations, or ulcerations of the skin, while at the same time the diagnostic criteria of an APLS are not fully met (e.g., 2 pregnancy losses or antibody titres in the lower range) (Table 2) [42].

### 2.3. Thyroid-Antibodies

Thyroid autoimmunity is associated with adverse pregnancy outcomes such as preterm delivery and pregnancy loss, as well as infertility [43]. The most common antibodies associated with RPL are anti-thyroid-peroxidase (anti-TPO) and anti-thyroglobulin (anti-TG) [44,45]. Two meta-analyses could associate thyroid autoimmunity with pregnancy loss and/or RPL [46,47]. However, later studies in RPL and IVF/ICSI patients did not confirm these associations [48,49]. Recently, in a large cohort of women with unexplained RPL (*n* = 825, with *n* = 139 being anti-TPO positive), a positive TPO antibody was predictive for a reduced LBR rate. Therapy with thyroxine increases LBR and thus supports the recommendation for screening for thyroid disorders [50]. As TPO antibodies are correlated with a two-fold increased risk of progression of subclinical hypothyroidism, biochemically defined as elevated thyroid-stimulating hormone (TSH) combined with normal thyroxine (T4) level, subclinical hypothyroidism can be seen as a mild form of thyroid failure caused by an autoimmune thyroid disorder [51,52].

The guidelines confirm the association of thyroid dysfunction and the risk of miscarriage and recommend screening for thyroid disorders and thyroid autoantibodies. The guideline of the ASRM, as well as the DGGG/OEGGG/SGGG and ESHRE, indicate an emerging consensus that TSH values >2.5 mU/L, should be considered as an abnormal result in RPL patients [5] (Table 2).

## 3. Alloimmunity

The alloimmune risk factors mentioned in the different guidelines are summarized in Table 1. Table 2 shows the suggested immunological diagnostics, that should be performed as current state of the art.

### 3.1. Natural Killer Cells

Natural killer (NK) cells belong to the innate immune system and are characterized by the expression of the surface marker CD56 [53]. Concerning reproductive immunology, two populations of NK cells are of special interest: peripheral NK cells (pNK, CD56^dim^CD16^bright^) and uterine NK cells (uNK, CD56^bright^CD16^dim^). PNK cells show a strong cytotoxic activity with antiviral and antineoplastic effects, whereas uNK cells are less cytotoxic and show a stronger immunomodulatory function than pNK cells [9,11]. UNK cells play an important role in trophoblast invasion and angiogenesis and represent about 70% of immune cells at the feto-maternal interface [54].

Due to controversial scientific evidence, there is no consensus for an explicit recommendation on analyzing pNK/uNK cells as well as testing NK cytotoxicity or NK activation so far [55]. Only the DGGG/OEGGG/SGGG recommends testing in case of pre-existing autoimmune disorders—that are not further defined. However, there is increasing evidence that NK cells may contribute to RPL [56]. A majority of studies, including one large meta-analysis, have shown increased levels of pNK cells in patients with RPL compared to healthy controls [9,56,57,58]. Furthermore, several studies emphasized the impact of previous live births on pNK concentrations as there is evidence of a different immune regulation regarding primary RPL (pRPL, patients that experienced no live birth yet) and secondary RPL patients (sRPL, patients with at least one live birth before the RPL) [59,60,61].

However, not only changes in the number of cells but also NK cell cytotoxic activity is being discussed to contribute to the pathophysiology of RPL [62]. Several studies report higher NK cytotoxicity in RPL patients before [63,64] and during [65,66,67] pregnancy as well as in pRPL patients [60]. However, a more recent study could not confirm the predictive value of pre-conceptional pNK cell activity in RPL patients [68]. In 2019, Sokolov et al. [69] emphasized that NK cell cytotoxic activity varies during the menstrual cycle that could explain certain alterations in previous studies.

UNK cells are likely to be more significantly involved in the process of embryo implantation than pNK cells [9]. So far, controversial results on uNK cells between women with RPL and controls have been reported [70,71,72]. Recently, increased numbers were shown particularly in patients with idiopathic RPL [57,73]. These differences may be due to lack of standardized uNK diagnostic (immunohistochemistry vs. flow cytometry), counting method (mm^2^ vs. percentages of stroma cells) as well as the establishment of a reference range in a fertile population [57,70,72,74,75].

### 3.2. Dendritic Cells

DC are controllers of the immune system by promoting not only the pro-inflammatory but also the tolerant/anti-inflammatory side of immune responses. Further, they are the only immune cells capable of activating naïve T-cells [76]. Due to these unique features, DCs are considered to be involved in the establishment of maternal tolerance during pregnancy. Moreover, silencing and establishment of tolerance go along with induction of Treg. Both Treg and DC are involved in the implantation process and maintenance of pregnancy [77,78]. Although DC comprises only approximately 1–2% of leukocytes in the endometrium, they have been shown to be key players in the human decidua [79]. Furthermore, most of the decidual DC in early pregnancy are found to be immature [80] and their presence in large numbers have been associated with the establishment of a healthy pregnancy [81]. In addition, inoculation by intravenous injection of syngeneic bone marrow-derived DC dramatically reduced the rate of spontaneous miscarriages (from 23.8% to 2.2%) in the murine abortion prone model CBA/J × DBA/2J [82].

ILT-4, a member of the immunoglobulin (Ig) gene superfamily, binds to HLA-G expressed on trophoblasts and involved in the contribution of immune tolerance at the feto-maternal interface. Recently, tolerized ILT-4 expressing DC have been found to be diminished peripheral blood and endometrial biopsies of patients with RPL [83]. The lower number of ILT-4 expressing DC correlated with a decreased number of Treg indicating a loss of tolerance induction in these patients. In contrast, large numbers of mature CD83^+^ DC might have a negative impact on implantation in RPL patients [82,83]. However, as the role of DC in RPL remains elusive, no guideline currently recommends analyzing DC.

### 3.3. Plasma Cells

Plasma cells (PC) develop from antigen-activated B-cells and secrete large quantities of antibodies in response to this antigen. A chronic inflammation of the endometrium (chronic endometritis, CE) is characterized by the presence of PC in the endometrial tissue. Plasma cells can be detected via immunohistochemistry using syndecan-1 (CD138) [84,85]. Irregular bleeding, pelvic pain, or dyspareunia can be symptoms of CE; however, in most cases, patients with a CE remain asymptomatic. CE could further negatively impact reproduction by altering uterine contractility, vascularization, decidualization, and autophagy [86]. Still, the cut-off level of CD138^+^ PC within the endometrium remains unclear. A wide range of diagnostic criteria is currently used: ≥1 PC/high power field (hpf), ≥1/section, ≥5/hpf, ≥1/10 hpf, ≥5/20 hpf, etc. [87]. Furthermore, there is no consensus on the timing of the biopsy within the menstrual cycle. While Kitaya et al. found no difference in identifying CE during the menstrual cycle [88], some studies showed a higher prevalence of CE in the proliferative phase compared to the secretory phase [89,90,91]. The underlying pathophysiological mechanism is likely to be an inflammatory reaction, which leads to an altered cytokine secretion [86,92]. CD68^+^ macrophages, CD83^+^ DC, CD8^+^ T cells, FOXp3^+^ Tregs [93], as well as uNK have shown to be elevated in CE [26]. CE can be identified in up to 58% of women suffering from RPL; however, more recent studies indicate a prevalence of around 10–20% [85,94,95]. The ESHRE guideline mentions CE; however, the authors conclude, that further research is needed before routine testing of RPL patients or treatment. The only guideline which recommends PC testing is the DGGG/OEGGG/SGGG guideline: in women with RPL, a biopsy of the endometrium can be performed to exclude a CE, and in the case of a CE, treatment with antibiotics can be recommended. Neither within the RCOG nor the ASRM guideline CE or PC are mentioned.

Since the stated inflammation is mainly caused by different bacteria [94], antibiotic treatment seems obvious. There has been no consensus on the possible treatment regime. However, a benefit of longer antibiotic courses was demonstrated [87]. First-line therapy with doxycycline for 14–21 days is a promising approach with cure-rates of up to 95% [96,97,98]. Still, a test of cure by performing a re-biopsy should be performed after completion. Second-line therapy with metronidazole and ciprofloxacin resulted in an overall cure rate of 99% in a study with patients suffering from recurrent implantation failure (RIF) [96].

### 3.4. Regulatory T-Cells

CD4^+^CD25^+^FoxP3^+^ regulatory T-cells (Tregs), a unique subpopulation of T-cells, play a crucial role in tolerance and prevention of autoimmunity and the success of allogeneic organ transplantations [99,100,101,102,103,104,105,106]. In pregnancy, Tregs are essential in tolerizing the maternal immune system towards the semi-allogeneic embryo. The modulation is either mediated via cell-to-cell contact or by secretion of immunosuppressive cytokines such as IL-10 and TGF-beta [107,108,109,110,111]. In humans, peripheral Tregs increase at the time of implantation and in early pregnancy, reaching peak levels in the second trimester and then decrease again after delivery [112,113,114,115].

Studies indicate lower peripheral and uterine Tregs in patients with RPL compared to healthy controls [116,117], which was recently confirmed in a large systematic review including 18 studies [118]. Besides RPL, maldistribution and functional impairment of Tregs have also been described in RIF and preeclampsia [119], highlighting their role in the earliest stages of pregnancy and placenta development. Mice, having a depletion of Tregs, showed a significant defect in implantation, which was reversed after an adoptive transfer of Tregs [120]. There is increasing evidence that pregnancy-associated hormones such as human chorionic gonadotropin are essential for immune balance in pregnancy, which is exerted, at least partly, by the expansion of Tregs [119].

Although it is not recommended in any guideline, analysis of Tregs might serve as potential targets for immunomodulatory treatments applied in randomized clinical trials.

## 4. Human Leukocyte Antigen System

A large number of genes located on chromosome 6 encodes the HLA. These genes are characterized by a broad polymorphism, which means that the HLA molecules of two people only match in a few places. A distinction is made between HLA class I (HLA A-G antigens) and HLA class II regions (HLA DR, DQ, and DP antigens) [121]. While a high level of HLA-sharing is a prerequisite for the immunological acceptance of allo-transplants, this does not seem to apply to the immunological interaction between the embryo and the mother. It is discussed that HLA-sharing impairs the maternal immune response that is necessary for implantation [122]. Further, it was shown that pregnancy losses occur more frequently when there is a match (sharing) in the HLA-C groups [123]. Increased frequencies of identical HLA-A and HLA-B alleles were associated with higher rates of RPL [124]. However, a large case-control study could not identify higher HLA-sharing in RPL couples [125]. Current guidelines do not recommend testing for HLA sharing.

Besides HLA-sharing, there is evidence that the functional outcome such as successful placentation depends on the interaction of the highly variable maternal killer immunoglobulin-like receptors (KIR) and fetal HLA-C genes [126,127,128]. HLA-C molecules can be divided into (1) HLA-C C1 acting as ligands for inhibitory KIRs, such as KIR2DL2/3 and (2) HLA-C C2 binding activating KIRs such as KIR2DL1 or KIR2DS1 [129]. Further, the maternal KIR genotypes can be categorized into type AA, resulting in a primarily inhibitory KIR-mediated NK cell response and type AB/BB holding multiple activating KIRs [15,130,131]. In women with pre-eclampsia as well as in RPL patients a certain combination of maternal genotype AA and increased parental HLA-C C2 molecules were detected, highlighting the genetic risk factor and the crucial balance of NK cell inhibition and activation for successful placentation [15,132]. Recently, a study suggested decreased HLA-C C1 ligands for KIR2DL2 result in an insufficient inhibition of NK cells and thereby contributing to RPL [130].

Within a cohort study sRPL occurs was more frequent in women with a first-born boy (adjusted OR 0.37; 95% CI 0.2–0.7) [133]. A following, prospective study (*n* = 358 sRPL patients) could identify the HLA class II alleles DRB1*15:01; −DQB1*05:01/05:02 and −DRB3*03:01 to be more prevalent in Scandinavian patients with a first-born boy that showed a lower LBR [134]. Based on this data, the ESHRE guideline states, that “HLA-DRB1*15:01 and HLA-DQB1*05:01/05:2 testing could be considered in Scandinavian women with sRPL after the birth of a boy, for prognostic purposes.

## 5. Therapeutic Options

We included an overview of the therapeutic options mentioned in the different guidelines in Table 3. Table 4 shows the suggested immunological therapies, that should be performed as current state of the art.

### 5.1. Corticosteroids

The supposed mechanism of action of corticosteroid-therapy in RPL is through promotion of the establishment of early pregnancy by suppression of uNK cells and improvement of trophoblast proliferation and invasion [136]. In the treatment of APLS, corticosteroids did not show a benefit, neither compared to aspirin, nor in combination with aspirin and heparin [137] with regard to live birth rate (LBR).

Glucocorticoid therapy on cycle days 1–21 in RPL patients with an increased uNK cell count significantly reduced the uNK levels [138]. However, other studies could only partially confirm these results, although some reported higher LBR in RPL patients treated with prednisolone [139,140,141]. Prednisolone treatment in patients with idiopathic RPL and elevated uNK cell numbers improved LBR as compared to controls (60% vs. 40%); however, the study population was very small (*n* = 20 patients vs. *n* = 20 controls) [140]. Still, due to adverse events and side effects of prednisolone, such as a higher risk of diabetes and hypertension in pregnancy and a significantly higher risk of preterm birth [142], the guidelines do not recommend the use of prednisolone until further studies are available. However, the use of corticosteroids can be reconsidered as there seems to be a dose-dependent effect, with adverse effects being less frequent in the case of a lower dosage of prednisolone (≤10 mg/kg daily) [143,144]. Recently, a study comparing the risk of preterm birth in patients with rheumatoid arthritis when treated with different dosages of prednisone showed an association between high dosage (≥10 mg/kg daily) and preterm birth, while lower doses (≤10 mg/kg daily) were not associated with preterm birth [145]. The DGGG/OEGGG/SGGG guideline suggests the therapeutic use of corticosteroids only for patients with preexisting auto-immune diseases. Just as with the ESHRE guideline, it points out the possibility of significant adverse events associated with the use of corticosteroids during pregnancy [146,147]. The need of further placebo-controlled randomized trials to identify specific risk factors is emphasized. The authors of the ASRM committee opinion and the RCOG guideline are in line and do not recommend the use of corticosteroids.

### 5.2. Intralipids

Intralipid, a 20% sterile fat emulsion containing soybean oil, phospholipids, glycerin, and water is suggested to be capable of modulating immune response by decreasing NK cytotoxicity, possibly mediated through short fatty acids, and inhibiting pro-inflammatory mediators, particularly Th1 cells [148,149]. However, the use of intralipid as an affordable and low-risk therapy strategy, its impact on LBR and implantation rate has not been consistently evaluated [150]. So far, only the DGGG/OEGGG/SGGG guideline considers intralipid as therapeutic options in RPL—however, not outside of clinical studies.

Several studies indicate a higher LBR in women with RPL treated with intralipid compared to those treated with IVIG [151,152]. However, Intralipid therapy has been investigated especially in women displaying elevated pNK cells and NK cytotoxicity: A recent meta-analysis including 3 studies reported higher LBR in women with elevated pNK cells (>20%, >12%, >19%) receiving intralipids compared to RPL patients without treatment (OR: 1.7; CI 95%; *p* = 0.02) [153]. This highlights the need to identify the patients most likely responding to an intralipid treatment [151,154]. However, not all studies could confirm the improvement on pregnancy outcomes in RPL patients by administering intralipids [150]. Still, comparing previous studies proves to be complex due to different outcomes such as LBR, clinical pregnancy rate, implantation rate, and various investigated groups (RPL, RIF, idiopathic, non-idiopathic, with or without biomarkers). Concerning safety, several studies and reviews observed no concerns [57,151,152,154,155].

### 5.3. IVIG

Nine randomized placebo-controlled trials and five randomized controlled treatment trials (RCTs) investigating intravenous immunoglobulin (IVIG) efficacy in women with RPL have been published with conflicting results [156,157,158,159,160,161,162,163,164,165,166]. In 2014, a Cochrane systematic review and meta-analysis found no significant beneficial effect of IVIG over placebo in improving the LBR in RPL patients [167]. Egerup et al. confirmed the results by the Cochrane analysis, but subgroup analysis showed that women with sRPL were more likely to obtain a potential beneficial effect from IVIG (RR for no live birth 0.77, 95% CI 0.58–1.02, *p* = 0.06) [168]. However, the same authors performed the so far largest trial on IVIG in sRPL patients and could not show an increase in LBR in sRPL patients treated with IVIG when compared to placebo [164].

Due to the controversial data on IVIG in RPL, no guideline recommends IVIG treatment in RPL. However, the ESHRE guideline comments extensively on this therapeutic option, expressing the need for further RCTs focusing on sRPL patients.

### 5.4. LMWH/ASS

There has been no proven effect of LDA or heparin on pregnancy rate for ANA-positive women [142,169].

All guidelines recommend treating APLS by administering LDA (75–100 mg daily) in combination with unfractionated/ low-molecular-weight heparin. Only the DGGG/OEGGG/SGGG and the ESHRE specify the beginning of the therapy: the heparin therapy should start with a positive pregnancy test. LDA treatment can start before conception according to the ESHRE, whereas starting with a positive pregnancy test is supported by the DGGG/OEGGG/SGGG. The latter also determines the end of therapy: LDA should be administered until gestational week 34+0, while heparin should be administered for up to six weeks post-partum. This also applies to “non-criteria” APLS, which is not mentioned in other guidelines. However, other publications question this strategy, as well as the cessation of therapy in gestational week 34+0, and suggest an even longer therapy. A recent study could show that the incidence of pregnancy loss in women with NC APLS was significantly lower in the group treated with low-molecular-weight heparin (LMWH) and low dose aspirin (LDA) compared to LDA alone, whereas a Cochrane review from 2020 concluded that heparin in combination with LDA may increase LBR, but further research is needed [36,170,171]. The European League Against Rheumatism (EULAR) differentiates the administration of LDA and/or heparin between OAPLS, NC-OAPLS, and thrombotic APLS: (1) Thrombotic APLS is recommended to be treated with LDA and heparin in therapeutic dosage during pregnancy; (2) if recurrent pregnancy complications occur in patients with OAPLS, increasing heparin to therapeutic dosage or addition of low dose prednisolone or hydroxychloroquine in the first trimester could be considered; (3) during pregnancy, treatment with LDA alone or in combination with heparin depending on the individual risk profile is recommended in patients with NC-OAPLS [135].

However, in 10–15% of OAPLS patients, a so-called refractory OAPLS occurs, characterized by persisting of poor obstetric outcome despite treatment and a higher rate of triple positivity as well as LAC compared to OAPLS [143,172]. In these patients, additional steroids, hydroxychloroquine, and TNF alpha-blocker in addition to LDA and LMWH treatment seem to be useful as a higher LBR has been described [143,173].

### 5.5. Further Immunotherapies

Several other potential immunotherapies, hardly mentioned in the guidelines such as TNF-blocker, lymphocyte immunotherapy (LIT), or granulocyte-colony-stimulating-factor (G-CSF) are in a debate to enhance LBR; however, the absence of standardized procedures to detect immunological disorders and small sample sizes impede the finding of evidence-based treatment options [9,174].

Within the therapy of LIT, several mechanisms, such as the production of anti-paternal antibodies, are suggested to promote a favorable immune environment for embryo implantation [175,176]. A meta-analysis reported a beneficial effect in RPL patients treated with LIT with a significant improvement in the LBR. Respecting safety, the authors conclude LIT as a valid treatment for idiopathic RPL patients, while a recently published study highlights the risk of an iatrogenic autoimmune disorder induced by preconceptional LIT therapy [175,177].

TNF-alpha blockers are used to decrease inflammation and therefore are suggested to be useful in patients with APLS-related RPL [178,179]. Especially in women with elevated Th1/Th2 profile high-potency TNF blockers such as adalimumab and etanercept in combination with LMWH showed a significantly higher LBR [180]. Alijotas-Reig et al. (2017) concluded that a theoretical potential of low embryo-fetal toxicity cannot be ruled out, although neither maternal nor fetal major adverse reactions have been reported so far.

Several studies reported a higher LBR in patients with unexplained RPL when treated with G-CSF compared to RPL patients receiving placebo caused by a rise of Treg cell concentrations and a reduction of NK-cell cytotoxicity [181,182,183,184]. However, a recent randomized controlled trial including 150 patients with unexplained RPL could not confirm the efficacy of recombinant human G-CSF [185].

## 6. Discussion

The treatment of couples with RPL confronts the physician with several challenges. A structured and standardized diagnostic procedure is mandatory to explain the scope of the diagnostics performed that, in the further course lead, to targeted therapies. However, different guideline recommendations obscure such clear strategies. After all, the timing to initiate diagnostic procedures differs significantly between different guidelines. As stated in the recommendation of the ASRM, diagnostics of the most common causes seems justified after two consecutive clinical pregnancies. The guideline of the ESHRE does also include two non-consecutive losses, whereas the RCOG and DGGG/OEGGG/SGGG guidelines speak of three consecutive pregnancy losses. It is therefore crucial, to establish a workflow that takes other circumstances into account. A recent meta-analysis could not show a difference in the prevalence of uterine anomalies or an APLS in women with two versus three pregnancy losses [186]. Another very recent study aiming to predict the chances of live birth in the following pregnancies after previous pregnancy losses was able to show a significant association of the pregnancy history and age with chances for live birth. However, these factors alone proved to be insufficient to be a robust determinant for live birth [187]. Therefore, an investigation of RPL risk factors should be commenced with consideration of the woman’s age, the number and details of the miscarriages that have already occurred, as well as on other factors, such as autoimmune disorders or fetal karyotyping.

Within this review, we could show that several immunological factors govern embryo implantation. Numerous studies could show alterations in immune cells or autoantibodies leading to a higher risk of RPL; however, performed in very heterogenous study groups and/or diverging methodologies. The absence of high-level evidence regarding these factors has hindered them from being part of guideline recommendations. This lack of evidence is partly attributed to the fact, that most of the immunological factors being a prerequisite for a successful pregnancy are yet to be unveiled as the complex process of implantation remains to be understood [188]. However, although the APLS is well evaluated in comparison to other immunological alterations in RPL, there is a lack of precise recommendations: none of the guidelines differentiates between OAPLS, refractory OAPLS, NC-OAPLS, thrombotic APLS comparable to the EULAR guidelines. This is possibly not due to a lack of evidence but attributed another effect: the year of publication of the different guidelines ranges from 2011 to 2018. Therefore, more recent findings such as a differentiation of different subtypes of an APLS or a CE, with increasing evidence of potential benefits when treated adequately in RPL patients, are not mentioned in older guidelines [17,85,87,189,190]. Ultimately, the differences in the recommendations can be traced back to the consensus process within the guideline work, which often results in newer diagnostic and therapeutic methods not being included in the recommendations.

The guideline of the ESHRE emphasizes the need for more research in the field of immunological therapies in RPL by highlighting a need to “study the effect of moderate dosages of prednisolone in RPL (preferably in large, controlled trials)” and “the effect of IVIG treatment in women with sRPL.” However, in daily practice, the health burden presented by many RPL couples might lead the clinicians towards more experimental therapies, rather than including them into randomized controlled trials.

## Figures and Tables

**Table 1:** Immunological diagnostics mentioned in different guidelines.

		DGGG/OEGGG/SGGG (2018)	ESHRE (2017)	ASRM (2012)	RCOG (2011)
Autoimmune Risk Factors	APLS	Testing for ACA, LAC and Anti-β2-glykoprotein I antibodies detected on 2 separate occasions at an interval of 12 weeks Testing for non-criteria APLS if clinical manifestations are present	Testing for ACA and LAC, Anti-β2-Glykoprotein I antibodies could be considered	Testing for ACA, LAC and Anti-β2-Glykoprotein I antibodies	Testing of ACA or LAC two times 12 weeks apart
	IgA antibodies Transglutaminase	Testing for IgA antibodies against Transglutaminase can be performed in women with a history of food sensitivity followed by biopsy if positive	---	---	---
	ANA	If elevated ANA titres are diagnosed in RPL patients, antibodies should be further differentiated (SS-A/RO and SS-B/ lupus anticoagulant (LAC) antibodies) to rule out a Sjögren’s syndrome or lupus erythematosus	Could be considered for explanatory purpose	Not recommended	---
	Thyroid Antibodies	An endocrine workup determining TSH levels is recommended in women with RPL. If TSH levels are found to be abnormal, T3, T4, and thyroid autoantibody concentrations must be determined	TPO-antibodies recommended followed by T4 testing in case of abnormal screening results	Not recommended if euthyroid	---
Alloimmune Risk Factors	Immune Cells	Only if evidence of a pre-existing autoimmune disorder	Only HLA-DQBI*05:01/05:2 could be considered in Scandinavian woman with sRPL Insufficient evidence to recommend NK cell testing Testing anti-HLA antibodies in women with RPL is not recommended	Circulating CD16 NK cell testing is not recommended Controversial scientific evidence for testing mucosal CD16 NK cells Controversial scientific evidence for HLA typing	Testing for pNK/ uNK cells should not be offered routinely in the investigation of recurrent miscarriage
	Chronic Endometritis	Evaluation of chronic endometritis by endometrial biopsy with analysis of CD 138-positive plasma-cells	Further studies on the subject are necessary	-	-

ACA = anti-cardiolipin antibodies; ANA = antinuclear antibodies; APLS = antiphospholipid syndrome; ASRM = American Society for Reproductive Medicine; CD16 = cluster of differentiation 16; DGGG/OEGGG/SGGG = German/Austrian/Swiss Society of Obstetrics and Gynecology; ESHRE = European Society of Human Reproduction and Embryology; HLA = human leukocyte antigen; IgA = immunoglobulin A; LAC = lupus anticoagulants; NK = natural killer cells; pNK = peripheral natural killer cells; RCOG = Royal College of Obstetricians and Gynaecologists; sRPL = secondary recurrent pregnancy loss; SS-A/RO = Sjögren’s-syndrome-related antigen A autoantibodies; SS-B/lupus anticoagulant = Sjögren’s-syndrome-related antigen B autoantibodies; T3 = triiodothyronine; T4 = thyroxine; TSH = thyroid-stimulating hormone; TPO = thyroid peroxidase; uNK = uterine natural killer cells.

**Table 2:** Suggested immunological standard diagnostics.

		Suggested Procedure
**Autoimmune Risk Factors**	**APLS**	ACA, LAC, Anti-β2-glykoprotein I antibodies Analysis should be performed at two separate occasions at an interval of 12 weeks Consider a non-criteria APLS, if clinical manifestations are present (renal microangiopathy, neurological disorders, cardiac manifestations, or ulcerations of the skin)
	**IgA Antibodies Transglutaminase**	IgA antibodies against Transglutaminase should only be analyzed in women with a history of food sensitivity followed by colon biopsy if antibodies positive
	**ANA**	Only ANA titres >1:160 are considered as positive If the ANA titres are elevated, antibodies should be further differentiated (SS-A/RO and SS-B/ lupus anticoagulant (LAC)antibodies) to rule out Sjögren’s syndrome or lupus erythematosus
	**Thyroid Antibodies**	TSH level should be analysed. If TSH levels are >2.5 mU/L, T3, T4 and thyroid autoantibody concentrations should be determined
**Alloimmune Risk Factors**	**Immune Cells**	Controversial scientific evidence for dendritic cells or regulatory T-cells Most data available for uNKs, is controversial and testing can only be recommended within studies
	**Chronic Endometritis**	Evaluation of chronic endometritis by endometrial biopsy with analysis of CD 138-positive plasma-cells

ACA = anti-cardiolipin antibodies; ANA = antinuclear antibodies; APLS = antiphospholipid syndrome; SS-A/RO = Sjögren’s-syndrome-related antigen A autoantibodies; SS-B/lupus anticoagulant = Sjögren’s-syndrome-related antigen B autoantibodies; T3 = triiodothyronine; T4 = thyroxine; TSH = thyroid-stimulating hormone; uNK = uterine natural killer cells.

**Table 3:** Therapeutic options mentioned in different guidelines concerning immunological alterations.

	DGGG/OEGGG/SGGG (2018)	ESHRE (2017)	ASRM (2012)	RCOG (2011)
APLS Therapy	Low dose aspirin plus unfractionated heparin or low molecular heparin starting with day of positive pregnancy test. Aspirin until GW 34+0, heparin 6 weeks post-partum (APLS and non-criteria APLS)	Low dose aspirin starting before conception plus prophylactic dose unfractionated heparin or low molecular heparin starting with a positive pregnancy test	Low dose aspirin and unfractionated heparin	Low dose aspirin plus heparin
Thyroid Antibodies	Thyroid hormone substitution therapy can be administered in woman with RPL and latent hypothyroidism i.e., TPO antibodies	There is insufficient evidence to support treatment with levothyroxine in euthyroid women with thyroid antibodies and RPL outside a clinical trial	---	---
Chronic Endometritis	Therapy of a chronic endometritis can be performed.	-	-	-
Immunomodulatory Therapy	Glucocorticoids only in clinical studies in women with pre-existing autoimmune disorder Therapies with IVIG, allogeneic lymphocyte transfer, lipid infusions or TNF-α-blockers should not be performed outside of clinical studies	Glucocorticoids are not recommended as treatments for unexplained RPL or RPL with selected immunological biomarkers IVIG are not recommended as a treatment of RPL There is insufficient evidence to recommend intralipid therapy for improving live birth rate in women with unexplained RPL. Heparin or low dose aspirin are not recommended to improve live birth rate in women with unexplained RPL	IVIG are not recommended for pRPL	Immune treatments should not be offered routinely to women with recurrent miscarriage outside formal research studies

APLS = antiphospholipid syndrome; ASRM = American Society for Reproductive Medicine; DGGG/OEGGG/SGGG = German/Austrian/Swiss Society of Obstetrics and Gynecology; ESHRE = European Society of Human Reproduction and Embryology; IVIG = intravenous immune globulin; pRPL = primary recurrent pregnancy loss; RCOG = Royal College of Obstetricians and Gynaecologists; TNF-α-blockers = tumor necrosis factor alpha blocker.

**Table 4:** Suggested immunological standard therapies.

Diagnose	Suggested Procedure
APLS Therapy	(1) Thrombotic APLS is recommended to be treated with LDA and heparin in therapeutic dosage during pregnancy (2) In case of refractory OAPLS, increasing heparin to therapeutic dosage or addition of low dose prednisolone or hydroxychloroquine in the first trimester could be considered (3) During pregnancy, treatment with LDA alone or in combination with heparin depending on the individual risk profile is recommended in patients with NC-OAPLS [135] Aspirin until GW 34+0, heparin 6 weeks post-partum (APLS and non-criteria APLS)
Thyroid Antibodies	Thyroid hormone substitution therapy can be administered in woman with RPL and latent hypothyroidism i.e., TPO antibodies
Chronic Endometritis	If detected, a chronic endometritis should be treated First line therapy with doxycycline 200 mg for 14 days. A test of cure should be performed after completion. Second line therapy with metronidazole and ciprofloxacin if test of cure is positive
Other Immunomodulatory Therapies	Glucocorticoids only in clinical studies in women with pre-existing autoimmune disorder therapies with IVIG, alogeneic lymphocyte transfer, lipid infusions or TNF-α-blockers can be considered, however not outside of clinical studies

APLS = antiphospholipid syndrome; IVIG = intravenous immune globulin; TNF-α-blocker = tumor necrosis factor alpha blocker.

## References

[1] WHO (1977). Recommended definitions, terminology and format for statistical tables related to the perinatal period and use of a new certificate for cause of perinatal deaths. Acta Obs. Gynecol. Scand..

[2] Practice Committee of the American Society for Reproductive Medicine (2008). Definitions of infertility and recurrent pregnancy loss. Fertil. Steril..

[3] Rai R., Regan L. (2006). Recurrent miscarriage. Lancet.

[4] Bender Atik R., Christiansen O.B., Elson J., Kolte A.M., Lewis S., Middeldorp S., Nelen W., Peramo B., Quenby S., Eshre Guideline Group on RPL (2018). Eshre guideline: Recurrent pregnancy loss. Hum. Reprod. Open.

[5] Practice Committee of the American Society for Reproductive Medicine (2012). Evaluation and treatment of recurrent pregnancy loss: A committee opinion. Fertil. Steril..

[6] Toth B., Wurfel W., Bohlmann M., Zschocke J., Rudnik-Schoneborn S., Nawroth F., Schleussner E., Rogenhofer N., Wischmann T., von Wolff M. (2018). Recurrent miscarriage: Diagnostic and therapeutic procedures. Guideline of the dggg, oeggg and sggg (s2k-level, awmf registry number 015/050). Geburtshilfe Frauenheilkd.

[7] Royal College of Obstetricians and Gynaecologists (2011). The investigation and treatment of couples with recurrent first-trimester and second-trimester miscarriage. Rcog Green Top. Guidel..

[8] Carrington B., Sacks G., Regan L. (2005). Recurrent miscarriage: Pathophysiology and outcome. Curr. Opin. Obs. Gynecol..

[9] Seshadri S., Sunkara S.K. (2014). Natural killer cells in female infertility and recurrent miscarriage: A systematic review and meta-analysis. Hum. Reprod. Update.

[10] Hanna J., Goldman-Wohl D., Hamani Y., Avraham I., Greenfield C., Natanson-Yaron S., Prus D., Cohen-Daniel L., Arnon T.I., Manaster I. (2006). Decidual nk cells regulate key developmental processes at the human fetal-maternal interface. Nat. Med..

[11] Tang A.W., Alfirevic Z., Quenby S. (2011). Natural killer cells and pregnancy outcomes in women with recurrent miscarriage and infertility: A systematic review. Hum. Reprod. (Oxf. Engl.).

[12] Tuckerman E., Mariee N., Prakash A., Li T.C., Laird S. (2010). Uterine natural killer cells in peri-implantation endometrium from women with repeated implantation failure after ivf. J. Reprod. Immunol..

[13] Leber A., Teles A., Zenclussen A.C. (2010). Regulatory t cells and their role in pregnancy. Am. J. Reprod. Immunol..

[14] Jin L.P., Chen Q.Y., Zhang T., Guo P.F., Li D.J. (2009). The cd4+cd25 bright regulatory t cells and ctla-4 expression in peripheral and decidual lymphocytes are down-regulated in human miscarriage. Clin. Immunol..

[15] Hiby S.E., Regan L., Lo W., Farrell L., Carrington M., Moffett A. (2008). Association of maternal killer-cell immunoglobulin-like receptors and parental hla-c genotypes with recurrent miscarriage. Hum. Reprod. (Oxf. Engl.).

[16] Hong Li Y., Marren A. (2018). Recurrent pregnancy loss: A summary of international evidence-based guidelines and practice. Aust J. Gen. Pr..

[17] Youssef A., Vermeulen N., Lashley E., Goddijn M., van der Hoorn M.L.P. (2019). Comparison and appraisal of (inter)national recurrent pregnancy loss guidelines. Reprod Biomed. Online.

[18] Ying Y., Zhong Y.P., Zhou C.Q., Xu Y.W., Wang Q., Li J., Shen X.T., Wu H.T. (2012). Antinuclear antibodies predicts a poor ivf-et outcome: Impaired egg and embryo development and reduced pregnancy rate. Immunol. Investig..

[19] Ying Y., Zhong Y.P., Zhou C.Q., Xu Y.W., Ding C.H., Wang Q., Li J., Shen X.T. (2013). A further exploration of the impact of antinuclear antibodies on in vitro fertilization-embryo transfer outcome. Am. J. Reprod. Immunol..

[20] D’Ippolito S., Ticconi C., Tersigni C., Garofalo S., Martino C., Lanzone A., Scambia G., Di Simone N. (2020). The pathogenic role of autoantibodies in recurrent pregnancy loss. Am. J. Reprod. Immunol..

[21] Veglia M., D’Ippolito S., Marana R., Di Nicuolo F., Castellani R., Bruno V., Fiorelli A., Ria F., Maulucci G., De Spirito M. (2015). Human igg antinuclear antibodies induce pregnancy loss in mice by increasing immune complex deposition in placental tissue: In vivo study. Am. J. Reprod. Immunol..

[22] Zeng M., Wen P., Duan J. (2019). Association of antinuclear antibody with clinical outcome of patients undergoing in vitro fertilization/intracytoplasmic sperm injection treatment: A meta-analysis. Am. J. Reprod. Immunol..

[23] Cavalcante M.B., Cavalcante C., Sarno M., da Silva A.C.B., Barini R. (2020). Antinuclear antibodies and recurrent miscarriage: Systematic review and meta-analysis. Am. J. Reprod. Immunol..

[24] Hefler-Frischmuth K., Walch K., Hefler L., Tempfer C., Grimm C. (2017). Serologic markers of autoimmunity in women with recurrent pregnancy loss. Am. J. Reprod. Immunol..

[25] Chen S., Yang G., Wu P., Sun Y., Dai F., He Y., Qian H., Liu Y., Shi G. (2020). Antinuclear antibodies positivity is a risk factor of recurrent pregnancy loss: A meta-analysis. Semin. Arthritis Rheum..

[26] Chen X., Liu Y., Zhao Y., Cheung W.C., Zhang T., Qi R., Chung J.P.W., Wang C.C., Li T.C. (2020). Association between chronic endometritis and uterine natural killer cell density in women with recurrent miscarriage: Clinical implications. J. Obs. Gynaecol Res..

[27] Harger J.H. (1992). Frequency of positive antinuclear antibody test results in women with recurrent spontaneous abortions. Am. J. Obs. Gynecol..

[28] Branch D.W., Gibson M., Silver R.M. (2010). Clinical practice. Recurrent miscarriage. N. Engl. J. Med..

[29] Vomstein K., Herzog A., Voss P., Feil K., Goeggl T., Strowitzki T., Toth B., Kuon R.J. (2020). Recurrent miscarriage is not associated with a higher prevalence of inherited and acquired thrombophilia. Am. J. Reprod. Immunol..

[30] Hughes G.R. (2011). Hughes syndrome (the antiphospholipid syndrome): A disease of our time. Inflammopharmacology.

[31] Esteve-Valverde E., Ferrer-Oliveras R., Alijotas-Reig J. (2016). Obstetric antiphospholipid syndrome. Rev. Clin. Esp..

[32] Taraborelli M., Reggia R., Dall’Ara F., Fredi M., Andreoli L., Gerosa M., Hoxha A., Massaro L., Tonello M., Costedoat-Chalumeau N. (2017). Longterm outcome of patients with primary antiphospholipid syndrome: A retrospective multicenter study. J. Rheumatol..

[33] Alijotas-Reig J., Esteve-Valverde E., Ferrer-Oliveras R., LLurba E., Ruffatti A., Tincani A., Lefkou E., Bertero M.T., Espinosa G., de Carolis S. (2018). Comparative study between obstetric antiphospholipid syndrome and obstetric morbidity related with antiphospholipid antibodies. Med. Clin..

[34] Alijotas-Reig J., Ferrer-Oliveras R., Group E.S. (2012). The european registry on obstetric antiphospholipid syndrome (euroaps): A preliminary first year report. Lupus.

[35] Arachchillage D.R., Machin S.J., Mackie I.J., Cohen H. (2015). Diagnosis and management of non-criteria obstetric antiphospholipid syndrome. Thromb Haemost.

[36] Alijotas-Reig J., Esteve-Valverde E., Ferrer-Oliveras R., Saez-Comet L., Lefkou E., Mekinian A., Belizna C., Ruffatti A., Hoxha A., Tincani A. (2020). Comparative study of obstetric antiphospholipid syndrome (oaps) and non-criteria obstetric aps (nc-oaps): Report of 1640 cases from the euroaps registry. Rheumatology (Oxf.).

[37] Perricone C., de Carolis C., Perricone R. (2012). Pregnancy and autoimmunity: A common problem. Best Pr. Res. Clin. Rheumatol..

[38] Abrahams V.M., Chamley L.W., Salmon J.E. (2017). Emerging treatment models in rheumatology: Antiphospholipid syndrome and pregnancy: Pathogenesis to translation. Arthritis Rheumatol..

[39] Girardi G., Berman J., Redecha P., Spruce L., Thurman J.M., Kraus D., Hollmann T.J., Casali P., Caroll M.C., Wetsel R.A. (2003). Complement c5a receptors and neutrophils mediate fetal injury in the antiphospholipid syndrome. J. Clin. Investig..

[40] Viall C.A., Chamley L.W. (2015). Histopathology in the placentae of women with antiphospholipid antibodies: A systematic review of the literature. Autoimmun Rev..

[41] Christiansen O.B. (2006). Evidence-based investigations and treatments of recurrent pregnancy loss. Curr. Opin. Obs. Gynecol..

[42] Miyakis S., Lockshin M.D., Atsumi T., Branch D.W., Brey R.L., Cervera R., Derksen R.H., PG D.E.G., Koike T., Meroni P.L. (2006). International consensus statement on an update of the classification criteria for definite antiphospholipid syndrome (aps). J. Thromb Haemost.

[43] Alexander E.K., Pearce E.N., Brent G.A., Brown R.S., Chen H., Dosiou C., Grobman W.A., Laurberg P., Lazarus J.H., Mandel S.J. (2017). 2017 guidelines of the american thyroid association for the diagnosis and management of thyroid disease during pregnancy and the postpartum. Thyroid.

[44] Ticconi C., Rotondi F., Veglia M., Pietropolli A., Bernardini S., Ria F., Caruso A., Di Simone N. (2010). Antinuclear autoantibodies in women with recurrent pregnancy loss. Am. J. Reprod. Immunol..

[45] Rushworth F.H., Backos M., Rai R., Chilcott I.T., Baxter N., Regan L. (2000). Prospective pregnancy outcome in untreated recurrent miscarriers with thyroid autoantibodies. Hum. Reprod. (Oxf. Engl.).

[46] Thangaratinam S., Tan A., Knox E., Kilby M.D., Franklyn J., Coomarasamy A. (2011). Association between thyroid autoantibodies and miscarriage and preterm birth: Meta-analysis of evidence. BMJ.

[47] Chen L., Hu R. (2011). Thyroid autoimmunity and miscarriage: A meta-analysis. Clin. Endocrinol. (Oxf.).

[48] Plowden T.C., Schisterman E.F., Sjaarda L.A., Zarek S.M., Perkins N.J., Silver R., Galai N., DeCherney A.H., Mumford S.L. (2016). Subclinical hypothyroidism and thyroid autoimmunity are not associated with fecundity, pregnancy loss, or live birth. J. Clin. Endocrinol. Metab..

[49] Unuane D., Velkeniers B., Deridder S., Bravenboer B., Tournaye H., De Brucker M. (2016). Impact of thyroid autoimmunity on cumulative delivery rates in in vitro fertilization/intracytoplasmic sperm injection patients. Fertil. Steril..

[50] Bliddal S., Feldt-Rasmussen U., Rasmussen A.K., Kolte A.M., Hilsted L.M., Christiansen O.B., Nielsen C.H., Nielsen H.S. (2019). Thyroid peroxidase antibodies and prospective live birth rate: A cohort study of women with recurrent pregnancy loss. Thyroid.

[51] Peeters R.P. (2017). Subclinical hypothyroidism. N. Engl. J. Med..

[52] Huber G., Staub J.J., Meier C., Mitrache C., Guglielmetti M., Huber P., Braverman L.E. (2002). Prospective study of the spontaneous course of subclinical hypothyroidism: Prognostic value of thyrotropin, thyroid reserve, and thyroid antibodies. J. Clin. Endocrinol. Metab..

[53] Robertson M.J., Ritz J. (1990). Biology and clinical relevance of human natural killer cells. Blood.

[54] Lash G.E., Bulmer J.N. (2011). Do uterine natural killer (unk) cells contribute to female reproductive disorders?. J. Reprod. Immunol..

[55] Guerrero B., Hassouneh F., Delgado E., Casado J.G., Tarazona R. (2020). Natural killer cells in recurrent miscarriage: An overview. J. Reprod. Immunol..

[56] Karami N., Boroujerdnia M.G., Nikbakht R., Khodadadi A. (2012). Enhancement of peripheral blood cd56(dim) cell and nk cell cytotoxicity in women with recurrent spontaneous abortion or in vitro fertilization failure. J. Reprod. Immunol..

[57] Kuon R.J., Muller F., Vomstein K., Weber M., Hudalla H., Rosner S., Strowitzki T., Markert U., Daniel V., Toth B. (2017). Pre-pregnancy levels of peripheral natural killer cells as markers for immunomodulatory treatment in patients with recurrent miscarriage. Arch. Immunol. Exp. (Warsz).

[58] King K., Smith S., Chapman M., Sacks G. (2010). Detailed analysis of peripheral blood natural killer (nk) cells in women with recurrent miscarriage. Hum. Reprod. (Oxf. Engl.).

[59] Kuon R.J., Vomstein K., Weber M., Muller F., Seitz C., Wallwiener S., Strowitzki T., Schleussner E., Markert U.R., Daniel V. (2017). The “killer cell story” in recurrent miscarriage: Association between activated peripheral lymphocytes and uterine natural killer cells. J. Reprod. Immunol..

[60] Shakhar K., Ben-Eliyahu S., Loewenthal R., Rosenne E., Carp H. (2003). Differences in number and activity of peripheral natural killer cells in primary versus secondary recurrent miscarriage. Fertil. Steril..

[61] Strobel L., Vomstein K., Kyvelidou C., Hofer-Tollinger S., Feil K., Kuon R.J., Ebner S., Troppmair J., Toth B. (2021). Different background: Natural killer cell profiles in secondary versus primary recurrent pregnancy loss. J. Clin. Med..

[62] Pandey M.K., Rani R., Agrawal S. (2005). An update in recurrent spontaneous abortion. Arch. Gynecol. Obstet..

[63] Aoki K., Kajiura S., Matsumoto Y., Ogasawara M., Okada S., Yagami Y., Gleicher N. (1995). Preconceptional natural-killer-cell activity as a predictor of miscarriage. Lancet.

[64] Matsubayashi H., Shida M., Kondo A., Suzuki T., Sugi T., Izumi S., Hosaka T., Makino T. (2005). Preconception peripheral natural killer cell activity as a predictor of pregnancy outcome in patients with unexplained infertility. Am. J. Reprod. Immunol..

[65] Emmer P.M., Nelen W.L., Steegers E.A., Hendriks J.C., Veerhoek M., Joosten I. (2000). Peripheral natural killer cytotoxicity and cd56(pos)cd16(pos) cells increase during early pregnancy in women with a history of recurrent spontaneous abortion. Hum. Reprod. (Oxf. Engl.).

[66] Yamada H., Morikawa M., Kato E.H., Shimada S., Kobashi G., Minakami H. (2003). Pre-conceptional natural killer cell activity and percentage as predictors of biochemical pregnancy and spontaneous abortion with normal chromosome karyotype. Am. J. Reprod. Immunol..

[67] Higuchi K., Aoki K., Kimbara T., Hosoi N., Yamamoto T., Okada H. (1995). Suppression of natural killer cell activity by monocytes following immunotherapy for recurrent spontaneous aborters. Am. J. Reprod. Immunol..

[68] Katano K., Suzuki S., Ozaki Y., Suzumori N., Kitaori T., Sugiura-Ogasawara M. (2013). Peripheral natural killer cell activity as a predictor of recurrent pregnancy loss: A large cohort study. Fertil. Steril..

[69] Sokolov D.I., Mikhailova V.A., Agnayeva A.O., Bazhenov D.O., Khokhlova E.V., Bespalova O.N., Gzgzyan A.M., Selkov S.A. (2019). Nk and trophoblast cells interaction: Cytotoxic activity on recurrent pregnancy loss. Gynecol. Endocrinol..

[70] Chen X., Mariee N., Jiang L., Liu Y., Wang C.C., Li T.C., Laird S. (2017). Measurement of uterine natural killer cell percentage in the periimplantation endometrium from fertile women and women with recurrent reproductive failure: Establishment of a reference range. Am. J. Obstet. Gynecol..

[71] Tuckerman E., Laird S.M., Prakash A., Li T.C. (2007). Prognostic value of the measurement of uterine natural killer cells in the endometrium of women with recurrent miscarriage. Hum. Reprod. (Oxf. Engl.).

[72] Clifford K., Flanagan A.M., Regan L. (1999). Endometrial cd56+ natural killer cells in women with recurrent miscarriage: A histomorphometric study. Hum. Reprod. (Oxf. Engl.).

[73] Gao Y., Wang P.L. (2015). Increased cd56(+) nk cells and enhanced th1 responses in human unexplained recurrent spontaneous abortion. Genet. Mol. Res..

[74] Michimata T., Ogasawara M.S., Tsuda H., Suzumori K., Aoki K., Sakai M., Fujimura M., Nagata K., Nakamura M., Saito S. (2002). Distributions of endometrial nk cells, b cells, t cells, and th2/tc2 cells fail to predict pregnancy outcome following recurrent abortion. Am. J. Reprod. Immunol..

[75] Tuckerman E., Laird S.M., Stewart R., Wells M., Li T.C. (2004). Markers of endometrial function in women with unexplained recurrent pregnancy loss: A comparison between morphologically normal and retarded endometrium. Hum. Reprod..

[76] Steinman R.M. (2012). Decisions about dendritic cells: Past, present, and future. Annu. Rev. Immunol..

[77] Teles A., Zenclussen A.C., Schumacher A. (2013). Regulatory t cells are baby’s best friends. Am. J. Reprod. Immunol..

[78] Craenmehr M.H., Heidt S., Eikmans M., Claas F.H. (2016). What is wrong with the regulatory t cells and foetomaternal tolerance in women with recurrent miscarriages?. HLA.

[79] Bulmer J.N., Williams P.J., Lash G.E. (2010). Immune cells in the placental bed. Int. J. Dev. Biol.

[80] Mansouri-Attia N., Oliveira L.J., Forde N., Fahey A.G., Browne J.A., Roche J.F., Sandra O., Reinaud P., Lonergan P., Fair T. (2012). Pivotal role for monocytes/macrophages and dendritic cells in maternal immune response to the developing embryo in cattle. Biol. Reprod..

[81] Askelund K., Liddell H.S., Zanderigo A.M., Fernando N.S., Khong T.Y., Stone P.R., Chamley L.W. (2004). Cd83(+)dendritic cells in the decidua of women with recurrent miscarriage and normal pregnancy. Placenta.

[82] Blois S., Alba Soto C.D., Olmos S., Chuluyan E., Gentile T., Arck P.C., Margni R.A. (2004). Therapy with dendritic cells influences the spontaneous resorption rate in the cba/j x dba/2j mouse model. Am. J. Reprod. Immunol..

[83] Liu S., Wei H., Li Y., Huang C., Lian R., Xu J., Chen L., Zeng Y. (2018). Downregulation of ilt4(+) dendritic cells in recurrent miscarriage and recurrent implantation failure. Am. J. Reprod. Immunol..

[84] Kitaya K., Matsubayashi H., Yamaguchi K., Nishiyama R., Takaya Y., Ishikawa T., Yasuo T., Yamada H. (2016). Chronic endometritis: Potential cause of infertility and obstetric and neonatal complications. Am. J. Reprod. Immunol..

[85] McQueen D.B., Perfetto C.O., Hazard F.K., Lathi R.B. (2015). Pregnancy outcomes in women with chronic endometritis and recurrent pregnancy loss. Fertil. Steril..

[86] Buzzaccarini G., Vitagliano A., Andrisani A., Santarsiero C.M., Cicinelli R., Nardelli C., Ambrosini G., Cicinelli E. (2020). Chronic endometritis and altered embryo implantation: A unified pathophysiological theory from a literature systematic review. J. Assist. Reprod. Genet..

[87] Huang W., Liu B., He Y., Xie Y., Liang T., Bi Y., Yuan L., Qin A., Wang Y., Yang Y. (2020). Variation of diagnostic criteria in women with chronic endometritis and its effect on reproductive outcomes: A systematic review and meta-analysis. J. Reprod. Immunol..

[88] Kitaya K., Yasuo T. (2011). Immunohistochemistrical and clinicopathological characterization of chronic endometritis. Am. J. Reprod. Immunol..

[89] Song D., Feng X., Zhang Q., Xia E., Xiao Y., Xie W., Li T.C. (2018). Prevalence and confounders of chronic endometritis in premenopausal women with abnormal bleeding or reproductive failure. Reprod Biomed. Online.

[90] Punnonen R., Lehtinen M., Teisala K., Aine R., Rantala I., Heinonen P.K., Miettinen A., Laine S., Paavonen J. (1989). The relation between serum sex steroid levels and plasma cell infiltrates in endometritis. Arch. Gynecol. Obstet..

[91] Eckert L.O., Hawes S.E., Wolner-Hanssen P.K., Kiviat N.B., Wasserheit J.N., Paavonen J.A., Eschenbach D.A., Holmes K.K. (2002). Endometritis: The clinical-pathologic syndrome. Am. J. Obs. Gynecol.

[92] Wang W.J., Zhang H., Chen Z.Q., Zhang W., Liu X.M., Fang J.Y., Liu F.J., Kwak-Kim J. (2019). Endometrial tgf-beta, il-10, il-17 and autophagy are dysregulated in women with recurrent implantation failure with chronic endometritis. Reprod. Biol. Endocrinol..

[93] Li Y., Yu S., Huang C., Lian R., Chen C., Liu S., Li L., Diao L., Markert U.R., Zeng Y. (2020). Evaluation of peripheral and uterine immune status of chronic endometritis in patients with recurrent reproductive failure. Fertil. Steril..

[94] Cicinelli E., Matteo M., Tinelli R., Pinto V., Marinaccio M., Indraccolo U., De Ziegler D., Resta L. (2014). Chronic endometritis due to common bacteria is prevalent in women with recurrent miscarriage as confirmed by improved pregnancy outcome after antibiotic treatment. Reprod. Sci. (Thousand Oaks Calif.).

[95] Kitaya K. (2011). Prevalence of chronic endometritis in recurrent miscarriages. Fertil. Steril..

[96] Kitaya K., Matsubayashi H., Takaya Y., Nishiyama R., Yamaguchi K., Takeuchi T., Ishikawa T. (2017). Live birth rate following oral antibiotic treatment for chronic endometritis in infertile women with repeated implantation failure. Am. J. Reprod. Immunol..

[97] Johnston-MacAnanny E.B., Hartnett J., Engmann L.L., Nulsen J.C., Sanders M.M., Benadiva C.A. (2010). Chronic endometritis is a frequent finding in women with recurrent implantation failure after in vitro fertilization. Fertil. Steril..

[98] Freitag N., Pour S.J., Fehm T.N., Toth B., Markert U.R., Weber M., Togawa R., Kruessel J.S., Baston-Buest D.M., Bielfeld A.P. (2020). Are uterine natural killer and plasma cells in infertility patients associated with endometriosis, repeated implantation failure, or recurrent pregnancy loss?. Arch. Gynecol. Obstet..

[99] Bestard O., Cruzado J.M., Mestre M., Caldes A., Bas J., Carrera M., Torras J., Rama I., Moreso F., Seron D. (2007). Achieving donor-specific hyporesponsiveness is associated with foxp3+ regulatory t cell recruitment in human renal allograft infiltrates. J. Immunol..

[100] Yoshizawa A., Ito A., Li Y., Koshiba T., Sakaguchi S., Wood K.J., Tanaka K. (2005). The roles of cd25+cd4+ regulatory t cells in operational tolerance after living donor liver transplantation. Transplant. Proc..

[101] Waldmann H., Graca L., Cobbold S., Adams E., Tone M., Tone Y. (2004). Regulatory t cells and organ transplantation. Semin. Immunol..

[102] Adorini L., Penna G., Giarratana N., Uskokovic M. (2003). Tolerogenic dendritic cells induced by vitamin d receptor ligands enhance regulatory t cells inhibiting allograft rejection and autoimmune diseases. J. Cell. Biochem..

[103] Kingsley C.I., Karim M., Bushell A.R., Wood K.J. (2002). Cd25+cd4+ regulatory t cells prevent graft rejection: Ctla-4- and il-10-dependent immunoregulation of alloresponses. J. Immunol..

[104] Venken K., Hellings N., Broekmans T., Hensen K., Rummens J.L., Stinissen P. (2008). Natural naive cd4+cd25+cd127low regulatory t cell (treg) development and function are disturbed in multiple sclerosis patients: Recovery of memory treg homeostasis during disease progression. J. Immunol..

[105] Sakaguchi S. (2004). Naturally arising cd4+ regulatory t cells for immunologic self-tolerance and negative control of immune responses. Annu. Rev. Immunol..

[106] Sakaguchi S., Sakaguchi N., Asano M., Itoh M., Toda M. (1995). Immunologic self-tolerance maintained by activated t cells expressing il-2 receptor alpha-chains (cd25). Breakdown of a single mechanism of self-tolerance causes various autoimmune diseases. J. Immunol..

[107] Huber S., Schramm C., Lehr H.A., Mann A., Schmitt S., Becker C., Protschka M., Galle P.R., Neurath M.F., Blessing M. (2004). Cutting edge: Tgf-beta signaling is required for the in vivo expansion and immunosuppressive capacity of regulatory cd4+cd25+ t cells. J. Immunol..

[108] Groux H. (2003). Type 1 t-regulatory cells: Their role in the control of immune responses. Transplantation.

[109] Nagaeva O., Jonsson L., Mincheva-Nilsson L. (2002). Dominant il-10 and tgf-beta mrna expression in gammadeltat cells of human early pregnancy decidua suggests immunoregulatory potential. Am. J. Reprod. Immunol..

[110] Hara M., Kingsley C.I., Niimi M., Read S., Turvey S.E., Bushell A.R., Morris P.J., Powrie F., Wood K.J. (2001). Il-10 is required for regulatory t cells to mediate tolerance to alloantigens in vivo. J. Immunol..

[111] Josien R., Douillard P., Guillot C., Muschen M., Anegon I., Chetritt J., Menoret S., Vignes C., Soulillou J.P., Cuturi M.C. (1998). A critical role for transforming growth factor-beta in donor transfusion-induced allograft tolerance. J. Clin. Investig..

[112] Somerset D.A., Zheng Y., Kilby M.D., Sansom D.M., Drayson M.T. (2004). Normal human pregnancy is associated with an elevation in the immune suppressive cd25+ cd4+ regulatory t-cell subset. Immunology.

[113] Sasaki Y., Sakai M., Miyazaki S., Higuma S., Shiozaki A., Saito S. (2004). Decidual and peripheral blood cd4+cd25+ regulatory t cells in early pregnancy subjects and spontaneous abortion cases. Mol. Hum. Reprod.

[114] Heikkinen J., Mottonen M., Alanen A., Lassila O. (2004). Phenotypic characterization of regulatory t cells in the human decidua. Clin. Exp. Immunol..

[115] Bao S.H., Wang X.P., De Lin Q., Wang W.J., Yin G.J., Qiu L.H. (2011). Decidual cd4+cd25+cd127dim/- regulatory t cells in patients with unexplained recurrent spontaneous miscarriage. Eur. J. Obstet. Gynecol. Reprod. Biol..

[116] Mei S., Tan J., Chen H., Chen Y., Zhang J. (2010). Changes of cd4+cd25high regulatory t cells and foxp3 expression in unexplained recurrent spontaneous abortion patients. Fertil. Steril..

[117] Inada K., Shima T., Nakashima A., Aoki K., Ito M., Saito S. (2013). Characterization of regulatory t cells in decidua of miscarriage cases with abnormal or normal fetal chromosomal content. J. Reprod. Immunol..

[118] Keller C.C., Eikmans M., van der Hoorn M.P., Lashley L. (2020). Recurrent miscarriages and the association with regulatory t cells; a systematic review. J. Reprod. Immunol..

[119] Huang N., Chi H., Qiao J. (2020). Role of regulatory t cells in regulating fetal-maternal immune tolerance in healthy pregnancies and reproductive diseases. Front. Immunol..

[120] Heitmann R.J., Weitzel R.P., Feng Y., Segars J.H., Tisdale J.F., Wolff E.F. (2017). Maternal t regulatory cell depletion impairs embryo implantation which can be corrected with adoptive t regulatory cell transfer. Reprod. Sci. (Thousand OaksCalif.).

[121] Christiansen O.B. (1996). A fresh look at the causes and treatments of recurrent miscarriage, especially its immunological aspects. Hum. Reprod. Update.

[122] Moghraby J.S., Tamim H., Anacan V., Al Khalaf H., Moghraby S.A. (2010). Hla sharing among couples appears unrelated to idiopathic recurrent fetal loss in saudi arabia. Hum. Reprod. (Oxf. Engl.).

[123] Beydoun H., Saftlas A.F. (2005). Association of human leucocyte antigen sharing with recurrent spontaneous abortions. Tissue Antigens.

[124] Christiansen O.B., Riisom K., Lauritsen J.G., Grunnet N., Jersild C. (1989). Association of maternal hla haplotypes with recurrent spontaneous abortions. Tissue Antigens.

[125] Aruna M., Nagaraja T., Andal Bhaskar S., Tarakeswari S., Reddy A.G., Thangaraj K., Singh L., Reddy B.M. (2011). Novel alleles of hla-dq and -dr loci show association with recurrent miscarriages among south indian women. Hum. Reprod. (Oxf. Engl.).

[126] Hiby S.E., Apps R., Sharkey A.M., Farrell L.E., Gardner L., Mulder A., Claas F.H., Walker J.J., Redman C.W., Morgan L. (2010). Maternal activating kirs protect against human reproductive failure mediated by fetal hla-c2. J. Clin. Investig..

[127] Moffett A., Chazara O., Colucci F., Johnson M.H. (2016). Variation of maternal kir and fetal hla-c genes in reproductive failure: Too early for clinical intervention. Reprod. Biomed. Online.

[128] Thielens A., Vivier E., Romagné F. (2012). Nk cell mhc class i specific receptors (kir): From biology to clinical intervention. Curr. Opin. Immunol..

[129] Parham P. (2005). Mhc class i molecules and kirs in human history, health and survival. Nat. Rev. Immunol..

[130] Yang X., Yang E., Wang W.-J., He Q., Jubiz G., Katukurundage D., Dambaeva S., Beaman K., Kwak-Kim J. (2020). Decreased hla-c1 alleles in couples of kir2dl2 positive women with recurrent pregnancy loss. J. Reprod. Immunol..

[131] Bashirova A.A., Martin M.P., McVicar D.W., Carrington M. (2006). The killer immunoglobulin-like receptor gene cluster: Tuning the genome for defense. Annu. Rev. Genom. Hum. Genet..

[132] Hiby S.E., Walker J.J., O’Shaughnessy K.M., Redman C.W., Carrington M., Trowsdale J., Moffett A. (2004). Combinations of maternal kir and fetal hla-c genes influence the risk of preeclampsia and reproductive success. J. Exp. Med..

[133] Nielsen H.S., Andersen A.M., Kolte A.M., Christiansen O.B. (2008). A firstborn boy is suggestive of a strong prognostic factor in secondary recurrent miscarriage: A confirmatory study. Fertil. Steril..

[134] Nielsen H.S., Steffensen R., Varming K., Van Halteren A.G., Spierings E., Ryder L.P., Goulmy E., Christiansen O.B. (2009). Association of hy-restricting hla class ii alleles with pregnancy outcome in patients with recurrent miscarriage subsequent to a firstborn boy. Hum. Mol. Genet..

[135] Tektonidou M.G., Andreoli L., Limper M., Amoura Z., Cervera R., Costedoat-Chalumeau N., Cuadrado M.J., Dörner T., Ferrer-Oliveras R., Hambly K. (2019). Eular recommendations for the management of antiphospholipid syndrome in adults. Ann. Rheum. Dis..

[136] Michael A.E., Papageorghiou A.T. (2008). Potential significance of physiological and pharmacological glucocorticoids in early pregnancy. Hum. Reprod. Update.

[137] Empson M., Lassere M., Craig J., Scott J. (2005). Prevention of recurrent miscarriage for women with antiphospholipid antibody or lupus anticoagulant. Cochrane Database Syst. Rev..

[138] Quenby S., Kalumbi C., Bates M., Farquharson R., Vince G. (2005). Prednisolone reduces preconceptual endometrial natural killer cells in women with recurrent miscarriage. Fertil. Steril..

[139] Fawzy M., Shokeir T., El-Tatongy M., Warda O., El-Refaiey A.A., Mosbah A. (2008). Treatment options and pregnancy outcome in women with idiopathic recurrent miscarriage: A randomized placebo-controlled study. Arch. Gynecol. Obstet..

[140] Gomaa M.F., Elkholy A.G., El-Said M.M., Abdel-Salam N.E. (2014). Combined oral prednisolone and heparin versus heparin: The effect on peripheral nk cells and clinical outcome in patients with unexplained recurrent miscarriage. A double-blind placebo randomized controlled trial. Arch. Gynecol. Obstet..

[141] Tempfer C.B., Kurz C., Bentz E.K., Unfried G., Walch K., Czizek U., Huber J.C. (2006). A combination treatment of prednisone, aspirin, folate, and progesterone in women with idiopathic recurrent miscarriage: A matched-pair study. Fertil. Steril..

[142] Laskin C.A., Bombardier C., Hannah M.E., Mandel F.P., Ritchie J.W., Farewell V., Farine D., Spitzer K., Fielding L., Soloninka C.A. (1997). Prednisone and aspirin in women with autoantibodies and unexplained recurrent fetal loss. N. Engl. J. Med..

[143] Bramham K., Thomas M., Nelson-Piercy C., Khamashta M., Hunt B.J. (2011). First-trimester low-dose prednisolone in refractory antiphospholipid antibody–related pregnancy loss. Blood.

[144] Palmsten K., Rolland M., Hebert M.F., Clowse M.E.B., Schatz M., Xu R., Chambers C.D. (2018). Patterns of prednisone use during pregnancy in women with rheumatoid arthritis: Daily and cumulative dose. Pharm. Drug Saf..

[145] Palmsten K., Bandoli G., Vazquez-Benitez G., Xi M., Johnson D.L., Xu R., Chambers C.D. (2020). Oral corticosteroid use during pregnancy and risk of preterm birth. Rheumatology (Oxf. Engl.).

[146] Gur C., Diav-Citrin O., Shechtman S., Arnon J., Ornoy A. (2004). Pregnancy outcome after first trimester exposure to corticosteroids: A prospective controlled study. Reprod. Toxicol..

[147] Hasbargen U., Reber D., Versmold H., Schulze A. (2001). Growth and development of children to 4 years of age after repeated antenatal steroid administration. Eur. J. Pediatrics.

[148] Roussev R.G., Acacio B., Ng S.C., Coulam C.B. (2008). Duration of intralipid’s suppressive effect on nk cell’s functional activity. Am. J. Reprod. Immunol..

[149] Granato D., Blum S., Rossle C., Le Boucher J., Malnoe A., Dutot G. (2000). Effects of parenteral lipid emulsions with different fatty acid composition on immune cell functions in vitro. Jpn. J. Parenter. Enter. Nutr..

[150] Martini A.E., Jasulaitis S., Fogg L.F., Uhler M.L., Hirshfeld-Cytron J.E. (2018). Evaluating the utility of intralipid infusion to improve live birth rates in patients with recurrent pregnancy loss or recurrent implantation failure. J. Hum. Reprod. Sci..

[151] Coulam C.B., Acacio B. (2012). Does immunotherapy for treatment of reproductive failure enhance live births?. Am. J. Reprod. Immunol..

[152] Meng L., Lin J., Chen L., Wang Z., Liu M., Liu Y., Chen X., Zhu L., Chen H., Zhang J. (2016). Effectiveness and potential mechanisms of intralipid in treating unexplained recurrent spontaneous abortion. Arch. Gynecol. Obstet..

[153] Placais L., Kolanska K., Kraiem Y.B., Cohen J., Suner L., Bornes M., Sedille L., Rosefort A., D’Argent E.M., Selleret L. (2020). Intralipid therapy for unexplained recurrent miscarriage and implantation failure: Case-series and literature review. Eur. J. Obstet. Gynecol. Reprod. Biol..

[154] Coulam C.B. (2020). Intralipid treatment for women with reproductive failures. Am. J. Reprod. Immunol..

[155] Ehrlich R., Hull M.L., Walkley J., Sacks G. (2019). Intralipid immunotherapy for repeated ivf failure. Fertil. Reprod..

[156] Perino A., Vassiliadis A., Vucetich A., Colacurci N., Menato G., Cignitti M., Semprini A.E. (1997). Short-term therapy for recurrent abortion using intravenous immunoglobulins: Results of a double-blind placebo-controlled italian study. Hum. Reprod. (Oxf. Engl.).

[157] Stephenson M.D., Dreher K., Houlihan E., Wu V. (1998). Prevention of unexplained recurrent spontaneous abortion using intravenous immunoglobulin: A prospective, randomized, double-blinded, placebo-controlled trial. Am. J. Reprod. Immunol..

[158] Jablonowska B., Selbing A., Palfi M., Ernerudh J., Kjellberg S., Lindton B. (1999). Prevention of recurrent spontaneous abortion by intravenous immunoglobulin: A double-blind placebo-controlled study. Hum. Reprod. (Oxf. Engl.).

[159] Christiansen O.B., Pedersen B., Rosgaard A., Husth M. (2002). A randomized, double-blind, placebo-controlled trial of intravenous immunoglobulin in the prevention of recurrent miscarriage: Evidence for a therapeutic effect in women with secondary recurrent miscarriage. Hum. Reprod. (Oxf. Engl.).

[160] Triolo G., Ferrante A., Ciccia F., Accardo-Palumbo A., Perino A., Castelli A., Giarratano A., Licata G. (2003). Randomized study of subcutaneous low molecular weight heparin plus aspirin versus intravenous immunoglobulin in the treatment of recurrent fetal loss associated with antiphospholipid antibodies. Arthritis Rheum.

[161] Mahmoud F., Diejomaoh M., Omu A., Abul H., Haines D. (2004). Effect of igg therapy on lymphocyte subpopulations in the peripheral blood of kuwaiti women experiencing recurrent pregnancy loss. Gynecol Obs. Investig..

[162] Dendrinos S., Sakkas E., Makrakis E. (2009). Low-molecular-weight heparin versus intravenous immunoglobulin for recurrent abortion associated with antiphospholipid antibody syndrome. Int. J. Gynaecol. Obstet. Off. Organ. Int. Fed. Gynaecol. Obstet..

[163] Stephenson M.D., Kutteh W.H., Purkiss S., Librach C., Schultz P., Houlihan E., Liao C. (2010). Intravenous immunoglobulin and idiopathic secondary recurrent miscarriage: A multicentered randomized placebo-controlled trial. Hum. Reprod. (Oxf. Engl.).

[164] Christiansen O.B., Larsen E.C., Egerup P., Lunoee L., Egestad L., Nielsen H.S. (2015). Intravenous immunoglobulin treatment for secondary recurrent miscarriage: A randomised, double-blind, placebo-controlled trial. BJOG.

[165] Kuroda K. (2018). Unexplained recurrent miscarriage: Introduction. Treatment Strategy for Unexplained Infertility and Recurrent Miscarriage.

[166] Daya S. (2020). Leucocyte immunotherapy for recurrent miscarriage. Recurrent Pregnancy Loss.

[167] Wong L.F., Porter T.F., Scott J.R. (2014). Immunotherapy for recurrent miscarriage. Cochrane Database Syst Rev..

[168] Egerup P., Lindschou J., Gluud C., Christiansen O.B., ImmuRe M.I.P.D.S.G. (2015). The effects of intravenous immunoglobulins in women with recurrent miscarriages: A systematic review of randomised trials with meta-analyses and trial sequential analyses including individual patient data. PLoS ONE.

[169] Stern C., Chamley L., Norris H., Hale L., Baker H.W. (2003). A randomized, double-blind, placebo-controlled trial of heparin and aspirin for women with in vitro fertilization implantation failure and antiphospholipid or antinuclear antibodies. Fertil. Steril..

[170] Hamulyak E.N., Scheres L.J., Marijnen M.C., Goddijn M., Middeldorp S. (2020). Aspirin or heparin or both for improving pregnancy outcomes in women with persistent antiphospholipid antibodies and recurrent pregnancy loss. Cochrane Database Syst. Rev..

[171] Li X., Deng X., Duan H., Zeng L., Zhou J., Liu C., Guo X., Liu X. (2021). Clinical features associated with pregnancy outcomes in women with positive antiphospholipid antibodies and previous adverse pregnancy outcomes: A real-world prospective study. Clin. Rheumatol..

[172] Mekinian A., Alijotas-Reig J., Carrat F., Costedoat-Chalumeau N., Ruffatti A., Lazzaroni M.G., Tabacco S., Maina A., Masseau A., Morel N. (2017). Refractory obstetrical antiphospholipid syndrome: Features, treatment and outcome in a european multicenter retrospective study. Autoimmun. Rev..

[173] Alijotas-Reig J., Esteve-Valverde E., Llurba E., Gris J.M. (2019). Treatment of refractory poor apl-related obstetric outcomes with tnf-alpha blockers: Maternal-fetal outcomes in a series of 18 cases. Semin. Arthritis Rheum..

[174] Achilli C., Duran-Retamal M., Saab W., Serhal P., Seshadri S. (2018). The role of immunotherapy in in vitro fertilization and recurrent pregnancy loss: A systematic review and meta-analysis. Fertil. Steril..

[175] Cavalcante M.B., Sarno M., Araujo Júnior E., Da Silva Costa F., Barini R. (2016). Lymphocyte immunotherapy in the treatment of recurrent miscarriage: Systematic review and meta-analysis. Arch. Gynecol. Obstet..

[176] Beer A.E., Quebbeman J.F., Ayers J.W.T., Haines R.F. (1981). Major histocompatibility complex antigens, maternal and paternal immune responses, and chronic habitual abortions in humans. Am. J. Obstet. Gynecol..

[177] Hou Y., Li J., Liu Q., Zhang L., Chen B., Li Y., Bian Y., Huang L., Qiao C. (2020). The optimal timing of immunotherapy may improve pregnancy outcome in women with unexplained recurrent pregnancy loss: A perspective follow-up study in northeastern china. Am. J. Reprod. Immunol..

[178] Liu J., Dong P., Jia N., Wen X., Luo L., Wang S., Li J. (2020). The expression of intracellular cytokines of decidual natural killer cells in unexplained recurrent pregnancy loss. J. Matern. Fetal Neonatal Med..

[179] Alijotas-Reig J., Esteve-Valverde E., Ferrer-Oliveras R., Llurba E., Gris J.M. (2017). Tumor necrosis factor-alpha and pregnancy: Focus on biologics. An updated and comprehensive review. Clin. Rev. Allergy Immunol..

[180] Winger E.E., Reed J.L. (2008). Treatment with tumor necrosis factor inhibitors and intravenous immunoglobulin improves live birth rates in women with recurrent spontaneous abortion. Am. J. Reprod. Immunol..

[181] Scarpellini F., Sbracia M. (2009). Use of granulocyte colony-stimulating factor for the treatment of unexplained recurrent miscarriage: A randomised controlled trial. Hum. Reprod..

[182] Santjohanser C., Knieper C., Franz C., Hirv K., Meri O., Schleyer M., Würfel W., Toth B. (2013). Granulocyte-colony stimulating factor as treatment option in patients with recurrent miscarriage. Arch. Immunol. Ther. Exp..

[183] Rutella S., Pierelli L., Bonanno G., Sica S., Ameglio F., Capoluongo E., Mariotti A., Scambia G., d’Onofrio G., Leone G. (2002). Role for granulocyte colony–stimulating factor in the generation of human t regulatory type 1 cells. Blood.

[184] Taga T., Kariya Y., Shimada M., Uchida A. (1993). Suppression of natural killer cell activity by granulocytes in patients with aplastic anemia: Role of granulocyte colony-stimulating factor. Immunol. Lett..

[185] Cavalcante M.B., Sarno M., Ricardo B. (2019). Recombinant human granulocyte–colony stimulating factor for all recurrent miscarriage patients or for a select group?. Hum. Reprod..

[186] van Dijk M.M., Kolte A.M., Limpens J., Kirk E., Quenby S., van Wely M., Goddijn M. (2020). Recurrent pregnancy loss: Diagnostic workup after two or three pregnancy losses? A systematic review of the literature and meta-analysis. Hum. Reprod. Update.

[187] Kolte A.M., Westergaard D., Lidegaard O., Brunak S., Nielsen H.S. (2021). Chance of Live Birth: A Nationwide, Registry-Based Cohort Study.

[188] Odendaal J., Quenby S., Sammaritano L., Macklon N., Branch D.W., Rosenwaks Z. (2019). Immunologic and rheumatologic causes and treatment of recurrent pregnancy loss: What is the evidence?. Fertil. Steril..

[189] Chen Y.Q., Fang R.L., Luo Y.N., Luo C.Q. (2016). Analysis of the diagnostic value of cd138 for chronic endometritis, the risk factors for the pathogenesis of chronic endometritis and the effect of chronic endometritis on pregnancy: A cohort study. BMC Womens Health.

[190] McQueen D.B., Bernardi L.A., Stephenson M.D. (2014). Chronic endometritis in women with recurrent early pregnancy loss and/or fetal demise. Fertil. Steril..

